# Monoclonal antibody inhibition of PAR2 reduces phenotype severity and pain in murine inflammatory bowel disease

**DOI:** 10.1097/PR9.0000000000001446

**Published:** 2026-04-20

**Authors:** Anne Ritoux, Laura J. Grundy, Pravallika Manjappa, Luke W. Paine, Jessica Neisen, John E. Linley, Ewan St. John Smith

**Affiliations:** a Department of Pharmacology, University of Cambridge, Cambridge, United Kingdom; b Neuroscience, BioPharmaceuticals R&D, AstraZeneca, Cambridge, United Kingdom; c Translational Science and Experimental Medicine, Research and Early Development, Respiratory and Immunology, BioPharmaceuticals R&D, AstraZeneca, Cambridge, United Kingdom

**Keywords:** Protease-activated receptor 2, Visceral pain, G protein–coupled receptor, Inflammatory pain, Colitis, Inflammatory bowel disease

## Abstract

**Introduction:**

Abdominal pain is a significant burden for those living with inflammatory bowel disease (IBD), representing a major unmet clinical need due to the scarcity of effective therapeutic options. The G protein–coupled receptor, protease-activated receptor 2 (PAR2), has emerged as a promising therapeutic target for visceral pain management in IBD.

**Objectives:**

We set out to determine signaling mechanisms deployed by PAR2 in the lumbar splanchnic nerve and whether inhibiting PAR2 with the mouse monoclonal antibody (mAb), PAR650097 (mPAR650097) provided therapeutic benefit in dextran sulfate sodium (DSS)-induced colitis.

**Methods:**

We first used ex vivo electrophysiological recordings of mouse lumbar splanchnic nerve to determine how stimulation of PAR2 alters afferent activity and the intracellular signaling mechanisms involved. Second, we used the DSS model of colitis in mice and determined how mPAR650097 altered disease activity, behavior, colon histology, and the activity of nociceptive circuitry.

**Results:**

Protease-activated receptor 2 stimulation in the colon activated visceral afferent fibers and sensitized them to mechanical and chemical stimuli. We found that endosomal internalization and protein kinase A/C signaling mediate both activating and sensitizing effects of PAR2 in visceral pain. We further show that inhibiting PAR2 with the mAb mPAR650097 reduces DSS-induced colitis severity and pain. mPAR650097 also reduced several colitis-induced pain correlates at key points along the pain-signaling axis, including peripheral nociceptive neuron signaling and sensitivity, expression of proinflammatory mediators, spinal cord signaling, and, fundamentally, behavior.

**Conclusion:**

These findings illustrate that mAb inhibition of PAR2 represents a promising approach to provide relief from abdominal pain in those living with IBD.

## Abstract 2

Protease-activated receptor 2 activation in the colon elicits pain-related signaling. Monoclonal antibody inhibition of protease-activated receptor 2 reduces murine colitis visceral hypersensitivity, pain-related behaviors, and phenotype severity.

## 1. Introduction

Globally, nearly 7 million people face shortened lifespan, increased risk of sepsis, and cancer from inflammatory bowel disease (IBD).^61^ IBD induces a debilitating and chronic inflammatory disorder of the gastrointestinal (GI) tract for which there are no cures. Patients with IBD seek health care to treat extreme and chronic abdominal pain, which significantly reduces their quality of life.^47^ However, current pain management strategies lack reproducible and widespread efficacy^4,9^ and often produce severe unwanted side effects.^4,9^ Consequently, there is an urgent need to find new therapeutic targets to relieve IBD-associated abdominal pain.

The G protein–coupled receptor protease-activated receptor 2 (PAR2) is a key player in visceral inflammatory pain. It is highly enriched in the GI tract of both mice^38^ and humans.^29^ Moreover, PAR2 and proteases that activate PAR2 have higher expression and activity in IBD patient biopsies^14,33^ and feces.^26^ In mice, intracolonic PAR2 activation, by proteases or peptide agonists such as 2-furoyl-LIGRLO-amide (2F), induces a colitis-like phenotype characterized by GI immune cell infiltration, mucosal damage, and increased epithelial permeability.^5,6,38,40^ Furthermore, PAR2 activation in the colon in vivo increases sensitivity to noxious mechanical and chemical stimuli.^5,10,27,30^ Mice with partial deletion, expression of protease-resistant PAR2, or mice treated with the PAR2-Gq-pathway selective antagonist GB88 experience milder colitis than wild-type animals.^20,25,40^ In vitro, PAR2 activation sensitizes cultured mouse sensory neurons^32^ and visceral afferent fibers.^27^

Despite evidence supporting a role for PAR2 in inflammatory pain, no PAR2 inhibitor is approved for use in humans. Promising preclinical results^34,35^ have led to the PAR2 inhibiting monoclonal antibody (mAb) MEDI0618 entering clinical trials for migraine therapy,^3^ but the effect of inhibiting PAR2 on IBD-associated visceral pain is not known.

Here, using ex vivo lumbar splanchnic nerve (LSN) electrophysiology recordings, we show that PAR2 activation in the colon increases basal action potential firing and causes hypersensitivity to mechanical and chemical stimuli, effects mediated by protein kinase A (PKA), protein kinase C (PKC), and endosomal internalization. In a mouse model of colitis, we further show that administering the PAR2 inhibitory mAb PAR650097 mouse IgG1 (mPAR650097) reduces several colitis-associated pain correlates, thus providing evidence supporting the use of mAb therapy inhibiting PAR2 to relieve IBD-associated visceral pain.

## 2. Materials and methods

### 2.1. Ethics

This work was conducted in accordance with the United Kingdom Animals (Scientific Procedures) Act 1986 Amendment Regulations 2012, under Project License PP5814995 approved by the University of Cambridge Animal Welfare and Ethical Review Body. We initially studied responses in tissue from male and female mice. Since the trend in responses observed were similar in both sexes, and to minimize variability in the dataset, only males were included in the PAR2 pathway inhibition LSN experiments. To assess therapeutic benefit of anti-PAR2 mAb treatment, we used a mild 1.5% dextran sulfate sodium (DSS) colitis model. We have shown that female mice are more resistant to this mild colitis model, with little difference in disease activity index (DAI) and histology compared with healthy females.^47^ Therefore, in line with 3 Rs guidelines, only male mice were used for DSS experiments to reduce the total number of animals used. Four to 5 adult (10- to 14-week-old) C57BL/6J mice (Envigo), Huntingdon, United Kingdom were kept in standard 49 × 10 × 12-cm cages with nesting material and a plastic shelter. For DSS experiments, animals were single housed. Mice were provided with food and water ad-libitum, in temperature (21°C) and humidity-controlled housing with a 12-hour light/dark cycle. All animals were humanely euthanized using a rising concentration of CO_2_, death was confirmed with cervical dislocation.

### 2.2. Antibody production

PAR650097 mIgG1 (mPAR650097) was produced and characterized as previously described for PAR650097 hIgG1^35^ and reformatted as a mouse IgG1. Briefly, IgG expression plasmids were derived from published vectors^49^ and engineered to include the Epstein–Barr virus origin of replication (OriP). Human vectors were adapted for mouse antibody production by introducing mouse IgG γ1 and λ constant regions into separate VH and VL vector backbones. polymerase chain reaction (PCR) primers were designed to amplify the human PAR650097 VH and VL regions with flanking restriction sites, enabling direct digestion-ligation into the corresponding mouse constant-region expression vectors. Final constructs were sequenced to confirm the PAR650097 variable regions remained unchanged, and thus, binding properties of PAR650097 should be retained in mPAR650097.

### 2.3. Dextran sulfate sodium–induced colitis

For in vivo and post-hoc analyses, experimenters were blinded to groups. Mice were randomized into 3 groups of equal average weights. Two groups were provided with drinking water supplemented with 1.5% DSS (40 kDa J63606.22, Thermo Fisher Scientific, Cambridge, United Kingdom) for 3 days after which the animals were returned to standard drinking water. Control mice were provided with standard drinking water throughout. After DSS treatment, animals received intraperitoneal injections: standard water control group received IgG isotype control, and DSS-treated groups receiving either IgG isotype control or mPAR650097, all at 50 mg/kg; antibodies provided by AstraZeneca. Mice were monitored daily, measuring weight loss, stool consistency, and blood content to calculate DAI (Table 1).^8,59^ After 6 days, mice were culled and tissue collected for post-hoc experiments.

**Table 1:** Disease activity index scoring criteria.

Score	Weight loss	Stool consistency	Blood in stool
0	None	Normal	None
1	1%–5%		
2	5%–10%	Very soft	Slight bleeding
3	10%–15%		
4	>15	Watery diarrhea	Gross bleeding

### 2.4. Behavior

Time spent digging was measured as previously described^7^: mice were placed in a test cage for 3 minutes, videos acquired (30 fps) using an iPhone 11 before returning mice to their home cage, and number burrows dug counted. Two blinded experimenters measured digging time using a stopwatch. Pixel motion analysis and location tracking were conducted using ezTrack.^48^ Behavioral experiments started after at least a week of habituation and were conducted in the animals' holding room. Animals were acclimatized to the testing cages and experimenter for 3 minutes daily from 3 days before testing. Tests were conducted 2 to 4 hours into the holding room light cycle, the same experimenters always being present.

### 2.5. Tissue collection

After euthanasia, spleens were removed and weighed, and distal colons with associated mesentery and neurovascular bundles were dissected as previously described^59^; colon length, anus to cecum, was measured. Luminal content was flushed with Krebs buffer (24 mM NaCl, 4.8 mM KCl, 1.3 mM NaH_2_PO_4_, 2.5 mM CaCl_2_, 1.2 mM MgSO_4_, 11.1 mM glucose, and 25 mM NaHCO_3_ in H_2_O). For the myeloperoxidase (MPO) assay, a proximal colon sample (1 cm from cecum) was snap-frozen in liquid nitrogen and stored at −80°C.

Spinal cords were extruded in ice-cold PBS using hydraulic pressure.^52^ Spines were bisected, and dorsal root ganglia (DRG) containing colon-innervating sensory neurons T12, T13, L5, and L6 were collected in ice-cold PBS.^23^

For histology, a distal colon sample (0.5 cm from the anus), DRG, and spinal cords were postfixed in 4% formaldehyde (Sigma-Aldrich, Haverhill, United Kingdom) at 4°C overnight. Samples were washed 3 times with PBS and cryopreserved in PBS containing 30% sucrose wt/vol at 4°C overnight before embedding in Shandon M-1 Embedding Matrix (Thermo Fisher Scientific, Cambridge, United Kingdom), snap-frozen in liquid nitrogen, and stored at −80°C.

### 2.6. Ex vivo lumbar splanchnic nerve afferent recordings

Skeletal muscle was stripped away and colons tied to either end of a cannula in a recording chamber. Colons were superfused luminally with a syringe pump (Harvard Apparatus, Cambridge, United Kingdom) at 100 μL/min and serosally at 7 mL/min with a peristaltic pump (Gilson, Dunstable, United Kingdom) with carboxygenated (95% O_2_, 5% CO_2_) Krebs buffer, maintained at 32 to 34°C by an in-line heater (Warner Instruments, Hamden, United States of America). Krebs buffer was supplemented with nifedipine (voltage-gated calcium channel blocker) and atropine (nonselective muscarinic receptor antagonist) at 10 μM to reduce smooth muscle contractions.^59^ Luminal pressure was recorded using a pressure transducer (Neurolog ModelNL 108).

The LSN was isolated, tissue in the neurovascular bundle above the iliac artery bifurcation removed, and aspirated into a borosilicate glass suction electrode, being left to stabilize for 30 minutes. Multiunit action-potential signals were amplified with a 5-kHz gain and bandpass filtered at 100 to 1300 Hz (Neurolog, Digitimer Ltd, Welwyn Garden City, United Kingdom). About 50-Hz noise was digitally filtered (Humbug, Digitimer Ltd, Welwyn Garden City, United Kingdom). The signal was then digitized at 20 kHz (Micro1401; Cambridge Electronic Design and displayed using Spike2 software [Cambridge Electronic Design, Cambridge, United Kingdom]).

Drugs were diluted from stock solutions into final volumes of 20 mL Krebs. PitNot2 (PN2, Ab6120688, Abcam, Cambridge, United Kingdom), PitStop2 (PS2, Ab120687, Abcam, Cambridge, United Kingdom), H-89 (AG-CR1-0002, AdipoGen, Buckingham, United Kingdom), GF109203X (GFX, HY-13867, Med Chem Express, Cambridge, United Kingdom), and 2F (3015, R&D Systems, Abingdon, United Kingdom) were applied to the colon mucosa via the luminal inflow. Approximately 1 µM capsaicin (M2028, Sigma-Aldrich, Haverhill, United Kingdom) and 1 mM cinnamaldehyde (8.02505, Merck Millipore, Watford United Kingdom) were added by serosal perfusion. Repeat drug applications were separated by a 30-minute washout period. Responses to drug application were assessed by the peak change in action potential firing rate after drug application; responses to distention were measured at 5 mm Hg intervals and compared with area under the curve.

### 2.7. Histological colon assessment

Colon cryosections (20 μm) were cut using a Leica CM3000 cryostat and mounted onto SuperFrost slides (Thermo Fisher Scientific, Cambridge, United Kingdom). Sections were stained with Gill hematoxylin III (Sigma-Aldrich, Haverhill, United Kingdom) and eosin (0.5% wt/vol; Acros Organics, Cambridge, United Kingdom) (H&E).^1,59^ Coverslips were mounted with glycerol and imaged using a NanoZoomer S360 (Hamamatsu, Welwyn Garden City, United Kingdom).

Histological scoring^51^ assessed colitis-associated damage and inflammation (Table 2), a blinded experimenter scoring 5 H&E-stained colon sections/mouse at least 100 μm apart.

**Table 2:** Histopathological scoring chart, which produces a maximum score of 12.

Score	Immune cell infiltrate		Epithelial damage	Mucosal architecture/atypia
	Mucosa	Submucosa		
0	Normal		No change	No change
1	Mild		Epithelial infiltrate	Simple hyperplasia without dysplasia
2	Moderate		Crypt abscesses, erosion	Low- and high-grade dysplasia
3	Severe		Ulcerations	Invasive carcinoma

### 2.8. Myeloperoxidase assay

Snap frozen proximal colon samples were thawed, dried, weighed, cut into 1-mm pieces, and 9 mL/g CTAB buffer (50 mM KH_2_PO_4_ and 13.7 mM hexadecyltrimethylammonium bromide) was added to each sample. Samples were homogenized with a sonic dismembrator (Thermo Fisher Scientific, Cambridge, United Kingdom) for 20 seconds then centrifuged at 18,000*g* for 15 minutes at 4°C. Then, 20 μL supernatant was added to 380 μL of either assay buffer (0.0005% H_2_O_2_, 50 mM KH_2_PO_4_ and 683.6 μM o-dianisidine) or control buffer (50 mM KH_2_PO_4_ and 683.6 μM o-dianisidine). All assay and control conditions were prepared in duplicate. Supernatants were incubated (37°C, 30-minutes) and reactions then heat inactivated (60°C, 10 minutes). Absorbance was read on a Clariostar plate reader at 460 nm (BMG Labtech, Aylesbury, United Kingdom).

One unit is defined as the amount of MPO present in 1 g of tissue that catalyzes the decomposition of 1 μmol H_2_O_2_ at 37°C for 30 minutes. The extinction coefficient of o-dianisidine is 11.3, the total volume was 0.3 mL, the sample weight volume ratio was 0.111 μL/mg, and sample volume was 0.02 mL.

$$MPO\;activity\;\left(\frac{U}{g}\right)=\frac{\left(\frac{Absorbance\;assay-Absorbance\;control}{Extinction\;coefficient}\right)\times Total\;volume}{Sample\;weight\;volume\;ratio\times Sample\;volume}$$

### 2.9. Immunohistochemistry

Sections were rehydrated for 5 minutes in PBS, permeabilized with Triton-X 100 0.02% (vol/vol, Sigma-Aldrich, Haverhill, United Kingdom) for 5 minutes, and blocked in buffer containing donkey serum 5% (vol/vol, Sigma-Aldrich, Haverhill, United Kingdom), BSA 1% (vol/vol, Sigma-Aldrich, Haverhill, United Kingdom), and Triton-X 100 0.02% (vol/vol). Sections were incubated in primary antibodies overnight at 4°C. Cellular FOS (cFOS) labelled spinal cords were incubated in primary antibodies for 48 hours. Sections were incubated in 1:500 secondary antibodies for 2 hours. Primary and secondary antibodies were diluted in blocking buffer. Unless stated otherwise, incubations and washes were conducted at room temperature. Coverslips were mounted with MOWIOL (475904, Calbiochem, Gillingham, United Kingdom). Fluorescent and bright-field photomicrographs were acquired with an Olympus Bx51 microscope with Qicam camera.

### 2.10. Immunoreactivity quantification

Positively stained DRG neurons were quantified. Regions of interest (ROI) corresponding to neurons were segmented using Cellpose3,^58^ ROIs being visually inspected to correct any erroneous segmentation. A custom Python script measured ROI average pixel intensity and identified positively labelled cells. A ROI was considered positive if its average pixel intensity was higher than the average pixel intensity of the 3 “darkest” ROIs in the image plus 10 times their standard deviation.

Spinal cord dorsal horn lamina I cFOS positive cells were quantified using transient receptor potential vanilloid 1 (TRPV1) immunoreactivity to identify the lamina I region. Puncta corresponding to cFOS immunoreactive cells were counted in the TRPV1-defined lamina I. Bright-field photomicrographs were used to assign images to corresponding spinal segments in the Allen Brain Atlas.^21^ For each spinal segment in each mouse, counts for at least 2 spinal dorsal horn field of views were analyzed.

### 2.11. Ca^2+^-imaging

1321N1 parental cells (lacking PAR2 expression), 1321N1 cells stably expressing human PAR2 (1321N1-hPAR2, generated by AstraZeneca), and murine CMT-93 cells (endogenous PAR2 expression) were used to characterize mPAR650097. Cells were plated at 4000/well in growth medium on PDL-coated Greiner-Bio 384 Well CELLCOAT microplates. After 24 hours, supernatants were replaced with Fluo-4 No Wash Calcium Assay Kit (ThermoFisher, Waltham, MA). Ca^2+^ responses measured using the fluorescence imaging plate reader (FLIPR) Tetra system (Molecular Devices, San Jose, CA); excitation and emission wavelengths: 470 to 495 nm and 515 to 575 nm, respectively. When measuring the inhibitory concentration 50 (IC50), cells received PAR650097 mIgG or hIgG or isotype control antibodies, for 60 minutes at room temperature before addition of PAR2 activating matriptase^34^ (3946-SEB-010, R&D Systems, Abingdon, United Kingdom).

### 2.12. Statistical analysis

Statistical analyses were conducted using python libraries Pandas,^43^ NumPy,^18^ Statsmodels,^55^ and SciPy.^60^ Data were visualized using the python library Matplotlib^24^ Seaborn.^62^ Concentration–response curves, IC50, and effective concentration 50 (EC50) values were obtained using GraphPad Prism version 10.4. Single comparisons of independent samples were made using unpaired 2-tailed *t* tests, normality determined by Shapiro–Wilk test. Multiple comparisons were made using one-way analysis of variance (ANOVA) with post-hoc Bonferroni multiple comparisons test and 2-way repeated-measures ANOVA with post-hoc Tukey multiple comparison test. Nonparametric multiple comparisons used Kruskal–Wallis test and Wilcoxon signed-rank with Dunn correction. Individual raw data are available in data file S1, http://links.lww.com/PR9/A405.

## 3. Results

### 3.1. Colonic protease-activated receptor 2 stimulation activates sensory neurons via endosomal internalization, protein kinase A, and protein kinase C

**Figure 1.**
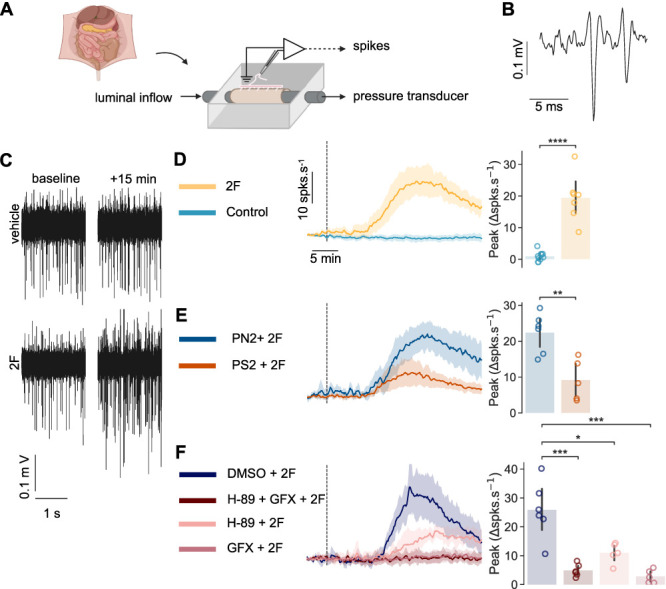
Colonic PAR2 activation by 2F elicits LSN responses that are mediated by endosomal internalization, PKA and PKC. (A) Experimental framework for LSN recordings showing a cannulated colon in a recording chamber with the LSN aspirated into a suction electrode. (B) Representative action potentials recorded from LSN afferent fiber prestimulation. (C and D) 100 µM 2F application elicits LSN responses illustrated by (C) action potential traces prestimulation and 15 minutes poststimulation and (D) change in LSN firing rate over time after stimulation via the luminal inflow (vertical dotted line). 2F-stimulation increased LSN firing (E and F). Timeline and peak change in firing rate after 100 µM 2F application (vertical dotted line) in tissue pretreated with (E) 50 µM PitStop2 (PS2) inhibitor for endosomal internalization or negative control PitNot2 (PN2) and (F) 100 µM H-89 dihydrochloride (H-89) and bisindolylmaleimide (GFX), PKA and PKC inhibitors or DMSO vehicle. (E) PS2 as well as separate and simultaneous pretreatment with H-89 and GFX reduced the peak response to 2F. (D and E) Independent samples *t* test comparing the peak change in firing rate (N = 5–9). (F) One-way ANOVA with post-hoc FDR-corrected independent samples *t* test (N = 5–6). *P < 0.05, **P < 0.01, ***P < 0.001 ****P < 0.0001. Data are presented as mean ± SD. LSN, lumbar splanchnic nerve; PAR2, protease-activated receptor 2; PKA, protein kinase A; PKC, protein kinase C.

To investigate PAR2's contribution to visceral afferent activity, we recorded from LSN afferent fiber bundles attached to cannulated colons (Fig. 1A); a response refers to the change in LSN action potential firing rate after stimulus application to the colon. Protease-activated receptor 2 was stimulated with the synthetic peptide agonist 2F^42^: 100 µM 2F elicited a 19.39 ± 7.29 spks·s^−1^ peak response in tissue from male mice (*P* = 1.015 × 10^−6^; Fig. 1B-D) and a 10.49 ± 6.76 spks·s^−1^ peak response in tissue from female mice (*P* = 1.95 × 10^−2^; Figure S1, http://links.lww.com/PR9/A405). Responses in tissue from female mice were less pronounced and more variable. Consequently, and because the aim was to inhibit PAR2 in a colitis model to which females are less susceptible,^47^ we focused on males for the rest of the study.

We next sought to determine how 2F modulates LSN activity. Endosomal internalization has been implicated in PAR2-mediated sustained trypsin-induced hyperexcitability of cultured DRG neurons and LSN mechanohypersensitivity.^27^ Therefore, we assessed if endosomal signaling contributed to 2F-induced LSN responses. Colons were pretreated for 30 minutes with the clathrin-mediated endocytosis inhibitor PS2 or the inactive control PN2. Compared with 50 µM PN2, inhibiting endosomal internalization with PS2 significantly reduced 2F-induced LSN responses (Peak Δspks·s^−1^, 21.66 ± 5.91 vs 9.18 ± 5.71, *P* = 3.790 × 10^−3^; Fig. 1E). Thus, PAR2 activation in the colon causes an immediate, transient increase in LSN firing that is mediated via endosomal signaling.

Lumbar splanchnic nerve mechanohypersensitivity and DRG hyperexcitability have been observed after PAR2 stimulation with proteases that activate distinct Gs- and Gq-coupled pathways.^27,39,64,65^ We, therefore, simultaneously inhibited PKA and PKC (100 µM H-89 and GFX, respectively) and no longer observed 2F-induced LSN responses. Separately, pretreatment with GFX also completely prevented the response to 2F, whereas pretreatment with H-89 significantly reduced 2F-induced LSN responses compared with the dimethyl sulfoxide (DMSO) vehicle control (Peak Δspks·s^−1^, GFX + H89, 4.88 ± 2.1, GFX 2.83 ± 2.32, H-89 10.99 ± 3.52 vs DMSO 25.85 ± 9.83, *F* = 19.49, *P*_*ANOVA*_ = 6.95 × 10^−6^, *P*_*simultaneous*_ = 4.614 × 10^−4^, P_H-89_ = 1.104 × 10^−2^, *P*_*GFX*_ = 6.699 × 10^−4^; Fig. 1F).

### 3.2. Colonic protease-activated receptor 2 stimulation sensitizes the lumbar splanchnic nerve to noxious stimuli

**Figure 2.**
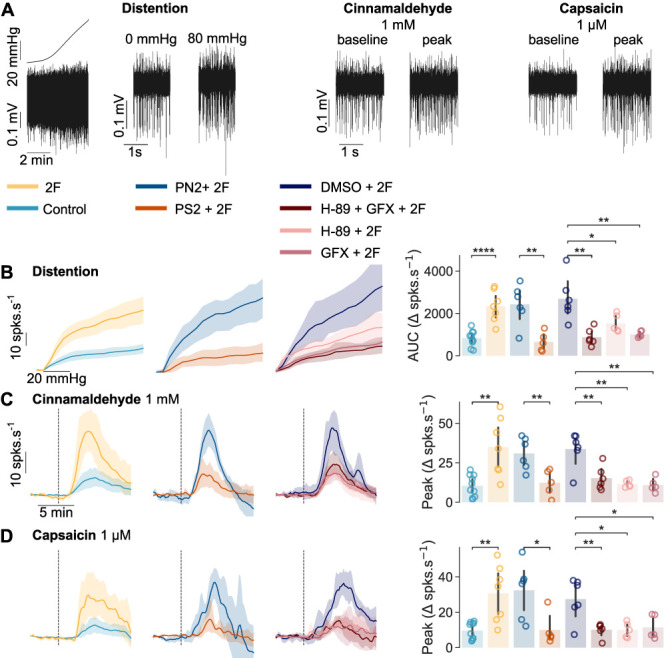
PAR2 activation in the colon by 2F sensitizes LSN responses to mechanical and chemical stimulation of the colon via endosomal internalization, PKA and PKC. LSN action potential firing after mechanical distention: gradually increasing intraluminal pressure from 0 to 80 mm Hg, and chemical application: 1 mM cinnamaldehyde and 1 µM capsaicin are illustrated in (A). (B) Responses to distention were quantified through the change in firing rate over increasing discrete pressure values. (C and D) Responses to chemical stimuli were quantified through the change in firing rate over time after application (vertical dotted line). 100 µM 2F stimulation, but not vehicle control, elicited sensitization of the LSN to (B) distention, (C) cinnamaldehyde, and (D) capsaicin. Pretreatment with PS2 before 2F stimulation reduced subsequent LSN responses to distention, cinnamaldehyde, and capsaicin compared with PN2. Pretreatment with H-89 and GFX, applied either simultaneously or individually, also reduced subsequent peak LSN responses to distention, cinnamaldehyde, and capsaicin compared with DMSO vehicle. (B–D) Independent samples *t* tests and one-way ANOVA with post-hoc FDR-corrected independent samples *t* test (N = 5–9). *P < 0.05, **P < 0.01, ****P < 0.0001. Data are presented as means ± SD. LSN, lumbar splanchnic nerve; PAR2, protease-activated receptor 2; PKA, protein kinase A; PKC, protein kinase C.

Inflammatory visceral pain is associated with sensory neuron hypersensitivity.^41,54^ Protease-activated receptor 2 activation sensitizes ion channels involved in visceral afferent function, including the capsaicin-, acid-, and heat-gated transient receptor potential vanilloid 1 ion channel,^11^ and the cinnamaldehyde-, acid-, and thermally sensitive TRP ankyrin 1 (TRPA1) ion channel.^12^ We stimulated colons with 1 mM cinnamaldehyde, 1 µM capsaicin, and distention (0–80 mm Hg) to probe LSN mechano- and chemosensitivity (Fig. 2A). Thirty-minute stimulation with 2F potentiated responses to mechanical distention (area under the curve [AUC], 2F 2339.78 ± 738.49 vs vehicle 822.74 ± 367.07; *P* = 4.793 × 10^−5^; Fig. 2B), cinnamaldehyde (Peak Δ spks·s^−1^, 34.977 ± 18.44 vs vehicle 10.47 ± 6.84; *P* = 1.475 × 10^−3^; Fig. 2C), and capsaicin (Peak Δspks·s^−1^, 30.69 ± 15.97 vs vehicle 9.61 ± 4.75; *P* = 1.210 ×10^−3^; Fig. 2D).

Because acute and sustained PAR2-mediated DRG hyperexcitability involves nonoverlapping signaling pathways mediated by PKC and endosomal internalization respectively,^27^ we sought to determine how these PAR2 signaling pathways contribute to sustained 2F-induced sensitization. Colons pretreated with PS2, as well as simultaneous or individual pretreatment with H-89 and GFX, prevented 2F-induced LSN sensitization to distention (Fig. 2B), cinnamaldehyde (Fig. 2C), and capsaicin (Fig. 2D and Table 3).

**Table 3:** Modulation of protease-activated receptor 2–sensitized responses to mechanical and chemical stimuli.

Pretreatment	Distention 0–80 mm Hg AUC Δ spks·s^−1^	Capsaicin 1 µM Peak Δ spks·s^−1^	Cinnamaldehyde 1 mM Peak Δ spks·s^−1^
PN2 50 µM	2430 ± 937.15	32.43 ± 15.69	12.40 ± 8.17
PS2 50 µM	652.96 ± 451.60 *P* = 3.85 × 10^−3^	9.99 ± 8.94 *P* = 1.99 × 10^−2^	30.94 ± 10.19 *P* = 9.62 × 10^−3^
DMSO	2692.42 ± 1043.62	27.46 ± 11.79	33.66 ± 11.11
H-89 + GFX 100 µM	873 ± 386.32 *P* = 2.50 × 10^−3^	10.12 ± 3.91 *P* = 6.56 × 10^−3^	15.22 ± 7.35 *P* = 6.87 × 10^−3^
H-89 100 µM	1526.24 ± 429 *P* = 4.52 ×10^−2^	10.02 ± 4.30 *P* = 1.24 × 10^−2^	11.55 ± 2.16 *P* = 1.86 × 10^−3^
GFX 100 µM	1011.55 ± 150.05 *P* = 6.32 ×10^−3^	11.47 ± 6.70 *P* = 2.53 × 10^−2^	10.88 ± 4.44 *P* = 2.05 × 10^−3^

Data are presented as means ± SD. Responses to distention, capsaicin, and cinnamaldehyde after PS2 + 2F or PN2 + 2F pretreatments are compared using independent samples *t* tests. Responses elicited after DMSO vehicle or H-89/GFX pretreatments with 2F stimulation were compared with one-way ANOVA (distention: *F* = 10.28, *P*_*ANOVA*_ = 4 × 10^−4^, cinnamaldehyde: *F* = 12.04, *P*_*ANOVA*_ = 1.47 × 10^−4^, capsaicin: *F* = 7.371, *P*_*ANOVA*_ = 2.01 × 10^−3^) followed by post-hoc FDR-corrected independent-samples *t* tests. (N = 5–9).

AUC, area under the curve.

### 3.3. mPAR650097 inhibiting of protease-activated receptor 2 reduces murine colitis severity

**Figure 3.**
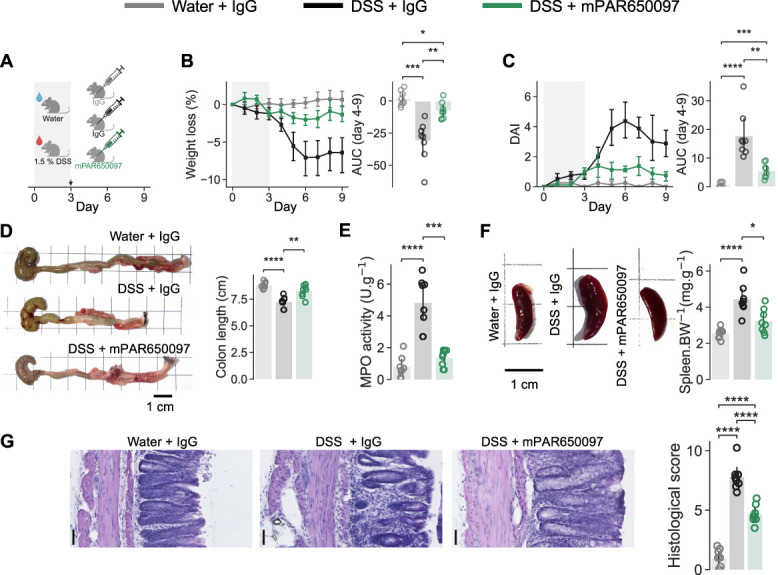
Therapeutic administration of mPAR650097 reduces DSS colitis severity. (A) Experimental timeline for the DSS colitis model and therapeutic dosing. Mice were given 1.5% DSS in drinking water or regular drinking water for 3 days followed by intraperitoneal injection of 50 mg/kg IgG or mPAR650097, they then received normal water for 6 days. Control mice received normal drinking water throughout and 50 mg/kg IgG intraperitoneal on day 3. Daily measurements of (B) weight and (C) DAI were used to assess phenotype progression. (D) Colon length with illustrative photographs, scale bars, 1 cm. (E) Colon myeloperoxidase activity, each point corresponds to the average of 3 experimental replicates. (F) Spleen weight normalized to the body weight was reduced in DSS-mPAR650097 mice compared with DSS-IgG mice with illustrative photographs, scale bar, 1 cm. (G) Blinded histopathology scores for ulceration and inflammation, illustrated by photomicrographs of H&E stained colon biopsies, scale bars, 50 µm. (B–G) One-way ANOVA with post-hoc Bonferroni multiple comparisons test (N = 8). *P < 0.05, **P < 0.01, ***P < 0.001, and ****P < 0.0001. Data are presented as means ± SD. DAI, disease activity index; DSS, dextran sulfate sodium.

Our data show that PAR2 activation stimulates and sensitizes colonic sensory nerves, and a plethora of evidence supports a role for proteases and PAR2 in IBD and visceral pain across species.^5,10,14,26,27,30^ Therefore, we sought to determine how the PAR2 inhibitory mAb mPAR650097, which potently inhibits PAR2 signaling (Figure S2, http://links.lww.com/PR9/A405),^35^ influences murine colitis; mPAR650097 avoids potential immunogenic responses to PAR650097 human IgG1 (hIgG1). We used the DSS-induced colitis model, mice receiving 1.5% DSS in their drinking water for 3-days. On day 3, mice were administered mPAR650097 (50 mg/kg) or IgG isotype control and returned to standard drinking water for 6 days; healthy control (HC) mice were maintained on standard drinking water and administered IgG on day 3 (Fig. 3A).

To assess the impact of mPAR650097, comparisons were made from day 4 onwards, i.e., 24 hours after mAb treatment. As expected, mice receiving DSS and control IgG (DSS-IgG) developed features similar to IBD, i.e., weight loss (AUC days 4–9, DSS-IgG −30.83 ± 15.67 vs water-IgG 2.02 ± 5.75, *F* = 21.141, *P*_*ANOVA*_ = 9.33 × 10^−6^, *P*_*HCvsDSS*_ _*IgG*_ = 2.08 × 10^−4^; Fig. 3B) and a worsening DAI (AUC days 4–9, DSS-IgG 17.79 ± 8.02 vs water-IgG 0.67 ± 0.84, *F =* 26.02, *P*_*ANOVA*_ = 2.07 × 10^−6^, *P*_*HCvsDSS*_ _*IgG*_ = 9.71 × 10^−5^; Fig. 3C). By contrast, DSS mice receiving mPAR650097 (DSS-mPAR650097) exhibited minimal weight loss and only a small, but still significant, increase in DAI (AUC days 4–9, weight loss −7.75 ± 6.67, *P*_*HCvsDSS mPAR650097*_ = 2.17 × 10^−3^, P_*DSS IgGvsDSS mPAR650097*_ = 5.48 × 10^−3^, DAI 5.61 ± 2.56; *P*_*HCvsDSS mPAR650097*_ = 4.04 × 10^−4^, *P*_*DSS IgGvsDSS mPAR650097*_ = 3.32 × 10^−3^; Fig. 3B-C).

Individuals with IBD experience a shortening of the small bowel,^46^ spleen enlargement,^31^ and increased fecal MPO activity.^17^ DSS-IgG mice had shorter colons (Fig. 3D), higher colon MPO activity (Fig. 3E), and larger spleens (Fig. 3F) compared with water-IgG and DSS-mPAR650097 mice; there were no significant differences between water-IgG and DSS-mPAR650097 groups. Compared with DSS-IgG mice, mPAR650097 administration resulted in a lower histological score, but it was higher than that of water-IgG mice (Fig. 3G and Table 4).

**Table 4:** *Post-hoc* colitis severity correlates.

Measurement	Water-IgG	DSS-IgG	DSS-mPAR650097
Colon length (cm) *F* = 22.46, *P*_*ANOVA*_ = 6.07 × 10^−6^	8.8 ± 0.31	7.23 ± 0.52	8.26 ± 0.57
	*P*_*HCvsDSS*_ _*IgG*_ = 1.00 × 10^−5^, *P*_*DSS IgGvsDSS mPAR650097*_ = 5.64 × 10^−3^		
MPO activity (U·g^−1^) *F* = 34.34, *P*_*ANOVA*_ = 2.40 × 10^−7^	0.77 ± 0.66	4.81 ± 1.62	1.34 ± 0.53
	*P*_*HCvsDSS*_ _*IgG*_ = 4.011 × 10^−5^, *P*_*DSS IgGvsDSS mPAR650097*_ = 1.47 × 10^−4^		
Spleen to body weight ratio (mg·g^−1^) *F* = 18.03, *P*_*ANOVA*_ = 2.76 × 10^−5^	2.55 ± 0.25	4.43 ± 0.82	3.22 ± 0.67
	*P*_*HCvsDSS*_ _*IgG*_ = 8.33 × 10^−5^, *P*_*DSS IgGvsDSS mPAR650097*_ = 1.85 × 10^−2^		
Histological score *F* = 113.40, *P*_*ANOVA*_ = 5.56 × 10^−1^	0.94 ± 0.79	7.79 ± 1.12	4.66 ± 0.78
	*P*_*HCvsDSS*_ _*IgG*_ = 3.38 × 10^−9^, *P*_*HCvsDSS*_ _*mPAR650097*_ = 5.29 × 10^−7^, *P*_*DSS IgGvsDSS mPAR650097*_ = 4.3910^−5^		

Data presented as means ± SD. Measurements were compared using one-way ANOVA with Bonferroni-corrected *t* tests (N = 8 per group).

DSS, dextran sulfate sodium; HC, healthy control.

### 3.4. mPAR650097 ameliorates colitis-associated behavioral changes

**Figure 4.**
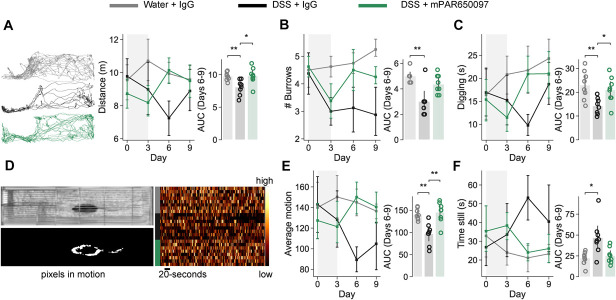
Administration of mPAR650097 ameliorates DSS colitis-induced changes in behavior. To assess the effect of the treatment on spontaneous behavior and activity, we measured how DSS-induced colitis affected unconditioned behaviors, locomotion, digging, and activity-state correlates on days 0, 3, 6, and 9. Locomotion was measured through the (A) distance travelled (m) across sessions illustrated by paths taken by example mice on day 6. Digging behavior was assessed through (B) the number of burrows and (C) time spent digging. Pixel motion was measured from 3-minute videos of animals freely moving illustrated in (D) example image of pixel motion segmentation for one frame (left) and pixel motion timeline heatmap from all animals on day 6 (right). From these, (E) average pixel motion and (F) time spent still were used to characterize activity state. (A–C, E and F) Grey shaded area corresponds to the DSS treatment, area under the curve (AUC) for days 6 to 9 (A, C, E and F) were compared using one-way ANOVA, and post-hoc Bonferroni multiple comparisons test (N = 8) (B) were compared using Kruskal–Wallis test and Wilcoxon signed-rank with Dunn correction *P < 0.05, **P < 0.01, ***P < 0.001, and ****P < 0.0001. Data are presented as means ± SD. DSS, dextran sulfate sodium.

Pain interferes with daily activities of patients with IBD^47^; we, thus measured how colitis affected unconditioned behaviors: locomotion and digging, and activity levels, and how these were modulated by mPAR650097. Post-treatment, DSS-IgG mice covered a shorter distance (Fig. 4A) and dug less (Fig. 4B-C) than DSS-mPAR650097 and water-IgG mice. In addition, pixel motion analysis shows that the DSS-IgG mice were overall less active than mice in the 2 other groups (Fig. 4D-F and Table 5).

**Table 5:** Colitis-induced pain behavioral correlates.

Measurement	Water IgG	DSS IgG	DSS mPAR650097
Distance travelled (m) *F = 6.96*, PANOVA = 4.80 × 10^−3^	9.758 ± 0.640	8.072 ± 1.158	9.822 ± 1.283
	*P*_*HCvsDSS IgG*_ = 8.64 × 10^−3^, *P*_*DSS IgGvsDSS*_ _*mPAR650097*_ = 3.75 × 10^−2^		
Number of burrows *H* = 10.70, *P* = 4.8 × 10^−3^	5 ± 0.463	3 ± 1.102	4.375 ± 0.744
	*P*_*HCvsDSS IgG*_ = 0.039		
Time spent digging (s) *F* = 6.27, *P*_*ANOVA*_ = 7.34 × 10^−3^	23.02 ± 6.14	14.25 ± 3.41	20.97 ± 5.61
	*P*_*HCvsDSS IgG*_ = 9.86 × 10^–3^, *P*_*DSS IgGvsDSS mPAR650097*_ = 3.50 ×10^−2^		
Time spent still (s) *F* = 7.19, *P*_*ANOVA*_ = 4.19 × 10^−3^	22.226 ± 7.829	46.815 ± 21.314	25.060 ± 9.428
	*P*_*HCvsDSS IgG*_ = 2.53 ×10^−2^		
Average pixel motion *F* = 12.987, *P*_*ANOVA*_ = 2.13 × 10^−3^	140.780 ± 12.690	96.975 ± 24.400	145.284 ± 23.656
	*P*_*HCvsDSS IgG*_ = 1.48 ×10^−3^, *P*_*DSS IgGvsDSS mPAR650097*_ = 3.79 × 10^−3^		

AUC values for days 4 to 9, data presented as means ± SD. Measurements were compared using one-way ANOVA with Bonferroni-corrected *t* tests (N = 8 per group), except for burrows that were compared using Kruskal–Wallis test and Wilcoxon signed-rank with Dunn correction (N = 8 per group).

AUC, area under the curve; DSS, dextran sulfate sodium; HC, healthy control.

### 3.5. mPAR650097 reduces dextran sulfate sodium–induced lumbar splanchnic nerve hypersensitivity

**Figure 5.**
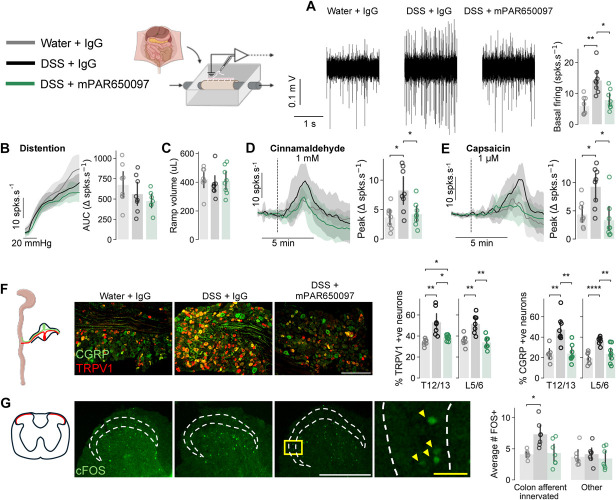
Administration of 50 mg/kg mPAR650097 alleviates 1.5% DSS-induced LSN fiber activity and sensitization. (A) Basal LSN action potential firing rate is reduced in DSS-mPAR650097 treated mice compared with DSS-IgG treated controls, representative neurograms for ex vivo LSN recordings (left) and average unstimulated firing rate (right). (B) Change in firing rate compared with baseline during colon distention. (C) Total volume required to reach 80 mm Hg indicating tissue compliance is unaffected (D and E). LSN responses to (D) 1 mM cinnamaldehyde and (E) 1 µM capsaicin, showing change in firing rate over time and peak change in firing rate after both stimuli. (F) Immunohistochemistry and quantification of neurons expressing TRPV1 and CGRP in thoracic T12/13 and lumbar L5/6 colon-innervating dorsal root ganglions, scale bar, 100 µm. (G) Average number of lamina I cFOS immunolabelled cells in spinal segments that receive inputs from colon-innervating visceral nociceptors (T12, T13, L5, L6, S1, S2), illustrated in photomicrographs, scale bar, 200 µm, yellow scale bar, 20 µm. (A–E) One- and (F and G) 2-way ANOVA with post-hoc Bonferroni multiple comparisons test (N = 7–8),*P < 0.05, **P < 0.01, ***P < 0.001, and ****P < 0.0001. Data are presented as means ± SD. CGRP, calcitonin gene–related peptide; DSS, dextran sulfate sodium; LSN, lumbar splanchnic nerve; TRPV1, transient receptor potential vanilloid 1.

Visceral hypersensitivity is characteristic of IBD^54^ and DSS-induced colitis.^41^ We, therefore, aimed to determine how mPAR650097 influences LSN activity postmortem. Approximately 1.5% DSS colitis resulted in a significantly higher basal LSN action potential firing rate compared with that of LSNs isolated from water-IgG mice, whereas the firing rate of LSN isolated from mPAR650097-DSS mice was comparable with that of healthy controls (spks·s^−1^, water-IgG 6.01 ± 2.92, DSS-IgG 14.25 ± 4.35, DSS-mPAR650097 7.95 ± 3.18, *F =* 11.84, *P*_*ANOVA*_ = 3.60 × 10^−4^, *P*_*HCvsDSS*_ _*IgG*_ = 1.67 × 10^−3^, *P*_*HCvsDSS mPAR650097*_ = 6.74 × 10^−1^, *P*_*DSS IgGvsDSS mPAR650097*_ = 1.56 × 10^−2^; Fig. 5A). Although no change in LSN mechanosensitivity or tissue compliance was observed between groups (Fig. 5B-C), DSS-IgG LSNs had significantly higher changes in peak firing rate in response to 1 mM cinnamaldehyde and 1 µM capsaicin compared with the same stimuli in water-IgG and DSS-mPAR650097 groups (cinnamaldehyde: Peak Δspks·s^−1^, water-IgG 3.71 ± 1.90, DSS-IgG 8.29 ± 3.63, DSS-mPAR650097 4.24 ± 2.06; *F =* 7.16, P_ANOVA_ = 4.26 × 10^−3^, *P*_*HCvsDSS*_ _*IgG*_ = 2.06 × 10^−2^, *P*_*DSS IgGvsDSS mPAR650097*_ = 4.78 × 10^−2^; Figure 5D; capsaicin: Peak Δspks·s^−1^, water-IgG 4.40 ± 2.33, DSS-IgG 9.29 ± 3.57, DSS-mPAR650097 3.36 ± 3.47; *F* = 7.98, *P*_*ANOVA*_ *=* 2.64 × 10^−3^, *P*_*HCvsDSS*_ _*IgG*_ = 1.76 × 10^−2^, *P*_*DSS IgGvsDSS mPAR650097*_ = 1.36 × 10^−2^; Fig. 5E). Dextran sulfate sodium–induced colitis causes sensitization to capsaicin^15^ and increased expression of TRPV1 and calcitonin gene–related peptide (CGRP) in colon-innervating DRG neurons.^16^ We, therefore, quantified TRPV1- and CGRP-expressing neurons in colon-innervating DRG (T12/13 and L5/6)^23^: DRG from DSS-IgG mice had a higher proportion of TRPV1- and CGRP-expressing neurons than DRG from both DSS-mPAR650097 and water-IgG mice (Fig. 5F and Table 6).

**Table 6:** Percentage of transient receptor potential vanilloid 1 and calcitonin gene–related peptide immunoreactive dorsal root ganglia neurons in colon-innervating dorsal root ganglia.

Immunoreactivity	Segment	Water-IgG	DSS-IgG	DSS-mPAR650097
TRPV1 *F*_*treatment*_ = 27.90 *P*_*ANOVA*_ = 3.01 × 10^−8^	T12/13	33.94 ± 3.09	52.96 ± 11.82	38.28 ± 2.69
		*P*_*HCvsDSS*_ _*IgG*_ = 7.254 × 10^−3^, *P*_*DSS IgGvsDSS mPAR650097*_ = 2.461 × 10^−2^		
	L5/6	35.88 ± 5.13	51.47 ± 8.92	33.91 ± 5.71
		*P*_*HCvsDSS*_ _*IgG*_ = 1.040 × 10^−2^, *P*_*DSS IgGvsDSS mPAR650097*_ = 2.958 × 10^−3^		
CGRP *F*_*treatment*_ = 26.34 *P*_*ANOVA*_ = 5.76 × 10^−8^	T12/13	23.41 ± 6.88	47.38 ± 13.45	23.88 ± 8.43
		*P*_*HCvsDSS*_ _*IgG*_ = 5.797 × 10^−3^, *P*_*DSS IgGvsDSS mPAR650097*_ = 5.474 × 10^−3^		
	L5/6	19.27 ± 5.93	36.75 ± 3.75	23.55 ± 8.80
		*P*_*HCvsDSS*_ _*IgG*_ = 1.534 × 10^−4^, *P*_*DSS IgGvsDSS mPAR650097*_ = 1.684 × 10^−2^		

Data presented as means ± SD. Measurements were compared using repeated-measures ANOVA with Bonferroni-corrected *t* tests (N = 7–8 per group).

DSS, dextran sulfate sodium; HC, healthy control; TRPV1, transient receptor potential vanilloid 1.

### 3.6. mPAR650097 reduces colitis-induced changes in spinal cord neurotransmission

To measure activation of spinal cord dorsal horn lamina I neurons, we quantified cFOS-positive nuclei within TRPV1-labeled regions across spinal cord segments T12-S4. Average cFOS + ve cell counts per section were grouped by spinal segments known to receive input from colon-innervating sensory neurons (T12, T13, L5, L6, S1, S2). Lamina I colonic circuitry relevant spinal segments had more cFOS-expressing (cFOS + ve) cells when isolated from DSS-IgG mice compared with when isolated from DSS-mPAR650097 and water-IgG mice (cFOS + ve cells per section, water-IgG 4.09 ± 0.64, DSS-IgG 7.31 ± 2.02, DSS-mPAR650097 4.31 ± 2.05, *F* = 5.90, *P*_*ANOVA*_ = 6.10 × 10^−2^, colon-innervated segments, *P*_*HCvsDSS*_ _*IgG*_ = 1.92 × 10^−2^, *P*_*DSS IgGvsDSS mPAR650097*_ = 3.18 × 10^−2^; Fig. 5E). By contrast, cFOS + ve lamina I neuron prevalence was comparable in spinal cord segments that do not receive colonic input (Fig. 5G-H).

## 4. Discussion

There is currently no cure or reliable efficacious therapy for pain management IBD. Our results support the concept of inhibiting PAR2 to provide relief of IBD-associated visceral pain. We find that colonic PAR2 stimulation activates visceral afferents and sensitizes them to noxious stimuli, results aligning with previous in vivo observations.^10,27,36,44^ We also show that PAR2-mediated LSN activation and sensitization involve endosomal internalization and PKA/PKC signaling. Although initial and sustained PAR2-mediated hyperexcitability of cultured DRG neurons require distinct signaling pathways, PKC vs endosomal internalization,^27,39^ our results suggest that multiple, and converging, signaling pathways subserve PAR2-mediated LSN activation and sensitization.

We cannot conclude if PAR2 activation at sensory nerve terminals causes nociceptor hyperexcitability, as previously hypothesized,^27^ or whether the effects result from PAR2 activation on non-neuronal cells, or a combination of both. In situ hybridization experiments show that PAR2 is expressed in only 2% to 4% of DRG neurons,^19,38^ and single-cell transcriptomics of colon-innervating DRG neurons failed to find high PAR2 expression,^23^ whereas PAR2 exhibits high expression in GI epithelial cells and circulating immune cells.^29^ Protease-activated receptor 2 activation in non-neuronal cells often causes proinflammatory cytokine release.^2,45,53,56^ Thus, colonic PAR2 activation might induce pain-related signaling directly at nerve terminals or indirectly via non-neuronal proinflammatory mediator release, or a combination of both; indeed, ever greater attention is being paid to non-neuronal cells in pain.^57^

Prophylactic PAR2 inhibition with a small molecule attenuates acute hapten-induced colitis severity.^40^ To improve translatability of our results, we conducted therapeutic intervention in a milder colitis model, 1.5% DSS drinking water rather than colorectal TNBS instillation. A limitation of our approach was collecting tissue at a single time point. Therefore, despite dosing with mPAR650097 3 days after colitis induction and observing therapeutic benefit for 6 days, we cannot distinguish between whether mPAR650097 treatment promotes recovery or prevents colitis aggravation. Experimental colitis studies using PAR2 deficient or inactive mice suggest a proregeneration role for PAR2 activation, which inhibits autophagy in colon epithelial cells.^20,22,33,63^ A separate study of multiple colitis models found that immune cell activation and recruitment is reduced in PAR2 deficient mice.^25^ These results are reconciled in a bimodular theory for PAR2 in colitis pathogenesis.^50^ Therefore, the reduced phenotype severity we observed might have arisen from inhibition of proinflammatory PAR2 signaling pathways. Nonetheless, a time-course study with mPAR650097 to assess improved recovery vs phenotype/pain prevention would be a logical next step.

Despite being unable to distinguish if therapeutic dosing with mPAR650097 promotes recovery or prevents colitis aggravation, we demonstrate that mPAR650097 reduces colitis-associated pain. Continuing our translational ambition, we largely focused on nonevoked visceral pain correlates. We have shown that decreased digging tracked with the percentage DSS administered and the extent of histological damage.^47^ Here, mPAR650097 improved colitis-depressed digging, locomotion and related activity state correlates (changes likely reflective of colitis-associated pain and/or malaise), reduced colitis-induced spinal cord lamina I neuron activation, and reduced colitis-induced LSN activity. One mechanism through which mPAR650097 could reduce colitis-associated pain is by preventing PAR2-induced nociceptive neuron sensitization. To support this, mPAR650097 reduced colitis-induced expression of mediators involved in hyperalgesia, TRPV1, and CGRP^13,36,44^ and reduced DSS-induced LSN sensitization to cinnamaldehyde and capsaicin. Our observation that DSS did not induce LSN sensitization to colonic distention is consistent with other observations that DSS does not sensitize visceromotor responses to rectal distention.^28,37^

In conclusion, our results provide strong evidence in support of the therapeutic benefit of inhibiting PAR2 using a mAb for the relief of abdominal inflammatory pain in IBD.

## Disclosures

P.M., J.N., and J.L. were employees of AstraZeneca at the time the work was conducted. A.R. and L.W.P. received PhD funding from AstraZeneca.

## Supplemental digital content

Supplemental digital content associated with this article can be found online at http://links.lww.com/PR9/A405.
