# Supplementary material for: Monoclonal antibody inhibition of PAR2 reduces phenotype severity and pain in murine inflammatory bowel disease
Source: Pain Rep. 2026 Apr 20;11(3):e1446. doi: 10.1097/PR9.0000000000001446 (PMC13098787; doi:10.1097/PR9.0000000000001446)
Supplement: Supplementary file 1 [file painreports-11-e1446-s001.pdf]

Supplementary Materials for
Monoclonal antibody inhibition of PAR2 reduces phenotype severity and pain in a murine inflammatory bowel disease model
Anne Ritoux *et al.*

The PDF includes:
Figs. S1 to S2

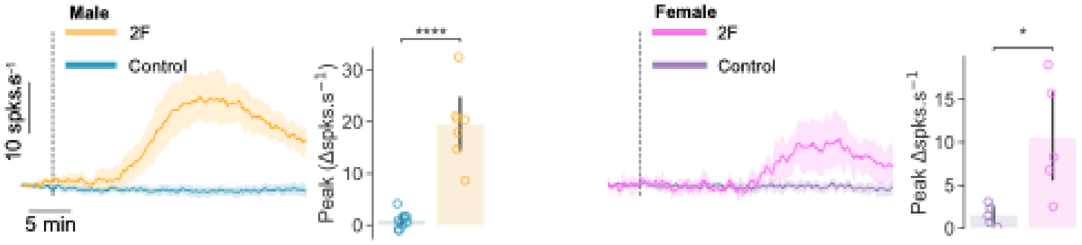

Fig. S1: PAR2 activation by 2F elicits a LSN response in tissue from female mice. Change in LSN firing rate over time following 100 µM 2F or vehicle application (vertical dotted line) in tissue from male (left) and female (right) mice. Male data are reproduced from figure 1, for ease of comparison. Independent samples t-test (N=5-9), **P* < 0.05. Data are presented as means ± SD.

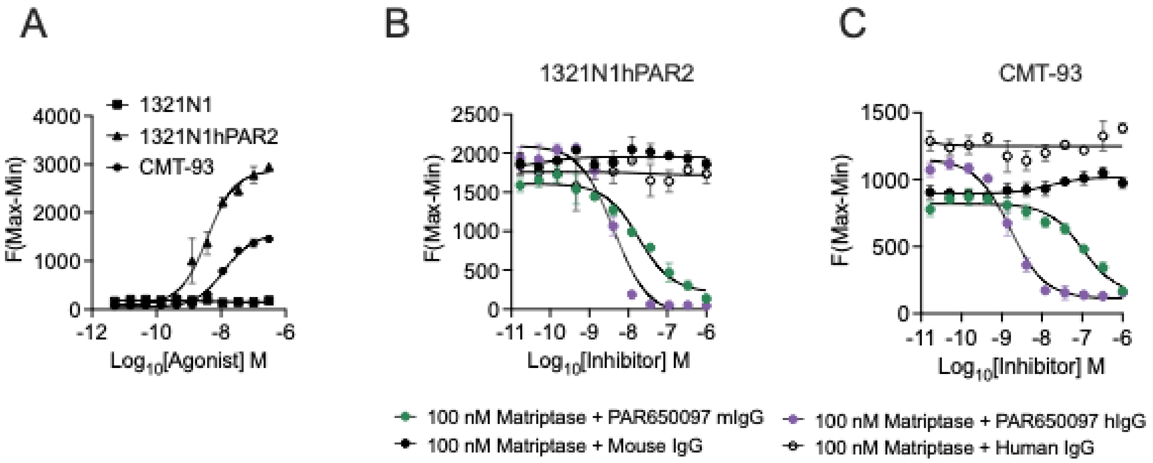

Fig.S2: mPAR650097 dose-dependently inhibits PAR2-dependent matriptase response observed with FLIPR measurements of cytosolic Ca^{2+}. (A) Matriptase produces dose-dependent Ca^{2+} transients in 1321N1hPAR2 (overexpressing human PAR2, EC50 1.07 nM) and in CMT-93 (natively expressing mouse PAR2, EC50 12.62 nM) but not in 1321N1 cells (lacking PAR2). (B-C) PAR650097 mIgG and hIgG dose-inhibitions of intracellular Ca^{2+} transients produced by 100 nM matriptase in (B) 1321N1hPAR2 cells ($IC50_{mPAR650097}$ 1.63 nM and $IC50_{PAR650097\ hIgG}$ 4.23 nM) (E) CMT-93 cells ($IC50_{mPAR650097}$100.52 nM and $IC50_{PAR650097\ hIgG}$ 1.67 nM). (n=3 experimental replicates) Data are presented as means ± SEM.
